# Continuous color tuning of single-fluorophore emission via polymerization-mediated through-space charge transfer

**DOI:** 10.1126/sciadv.abd1794

**Published:** 2021-04-07

**Authors:** Suiying Ye, Tian Tian, Andrew J. Christofferson, Sofia Erikson, Jakub Jagielski, Zhi Luo, Sudhir Kumar, Chih-Jen Shih, Jean-Christophe Leroux, Yinyin Bao

**Affiliations:** 1 Institute of Pharmaceutical Sciences, Department of Chemistry and Applied Biosciences, ETH Zurich, Vladimir-Prelog-Weg 1-5/10, 8093 Zurich, Switzerland.; 2 Institute for Chemical and Bioengineering, Department of Chemistry and Applied Biosciences, ETH Zurich, Vladimir-Prelog-Weg 1-5/10, 8093 Zurich, Switzerland.; 3 School of Science, College of Science, Engineering and Health, RMIT University, 124 La Trobe Street, Melbourne, Victoria 3000, Australia.

## Abstract

Tuning emission color of molecular fluorophores is of fundamental interest as it directly reflects the manipulation of excited states at the quantum mechanical level. Despite recent progress in molecular design and engineering on single fluorophores, a systematic methodology to obtain multicolor emission in aggregated or solid states, which gives rise to practical implications, remains scarce. In this study, we present a general strategy to continuously tune the emission color of a single-fluorophore aggregate by polymerization-mediated through-space charge transfer (TSCT). Using a library of well-defined styrenic donor (D) polymers grown from an acceptor (A) fluorophore by controlled radical polymerization, we found that the solid-state emission color can be fine-tuned by varying three molecular parameters: (i) the monomer substituent, (ii) the end groups of the polymer, and (iii) the polymer chain length. Experimental and theoretical investigations reveal that the color tunability originates from the structurally dependent TSCT process that regulates charge transfer energy.

## Teaser

Aggregation-induced through-space charge transfer can be used to tune single-fluorophore emission in simple polymer systems.

## INTRODUCTION

Among various light-emitting materials, organic/polymeric fluorescent materials are receiving increasing attention recently. They offer flexibility and diversity due to highly varied molecular design and material processing options (*1*–*6*). These materials exhibit great potential in various applications, such as chemical sensors (*1*), bioimaging (*2*), theranostics (*3*), organic light-emitting diodes (*4*), and secure printing (*5*, *6*). For basic research, as well as for practical use, it is of great interest to design light-emitting materials that can be fine-tuned regarding their emission spectrum, especially in their solid or aggregated states (*6*). The systems currently available rely on fluorophore derivatization (*7*, *8*) or covalent combination of different fluorophores in polymer chains to achieve distinct emission wavelengths (*9*–*11*). Specifically, by incorporating different donor (D)/acceptor (A) fluorophore pairs in one polymer, blue-to-red emission color can be achieved for the resulted through-space charge transfer (TSCT) polymers, owing to the varied D-A charge transfer (CT) strength (*12*–*14*). However, these strategies often require tedious and costly multistep organic syntheses. Supramolecular chemistry approaches (*15*–*17*) were also used to design color-tunable fluorescent materials, but their usability is limited as they require the combination of multiple chemical species that causes instability and complicates the fabrication of devices.

Recently, single organic dye molecules were found to display solid-state multicolor emission upon engineering their molecular conformation or packing mode (*18*–*22*). This is achieved by mechanical stimulation, thermal treatment, or solvent exchange, which then results in a CT process change. However, because of the strong dependency of the fluorescence output on the aggregation structure, it is challenging to achieve precise and reproducible engineering of the emission states with these physical methods. On the other hand, single-fluorophore–based multicolor emissive polymers attracted great attention, because of their ability to improve the stability/reproducibility of the solid-state emission and to simplify the fabrication of optoelectronic devices (*23*–*25*). For example, Zhang and coworkers (*23*) synthesized a series of single-fluorophore–based polyesters by cationic ring-opening polymerization. By increasing the chain length of the polymers, emissions from green to blue were observed. The color tunability was attributed to the local fluorophore-fluorophore interactions (*23*). Wan and colleagues (*24*) explored a polymerization-induced multicolor emission method based on Barbier hyperbranching polymerization. Likely due to the variation of intramolecular CT, the mechanism of this phenomenon needs further investigation. Although polymerization has the potential to manipulate molecular fluorescence, a systematic methodology to obtain multicolor emission from a single fluorophore in the aggregated state is still lacking.

Controlled radical polymerization (*26*–*28*), in particular, atom transfer radical polymerization (ATRP), has shown great versatility in preparing well-defined functional polymeric materials (*29*–*31*), including light-emitting polymers (*32*). Because of the fact that active initiating groups can be conveniently incorporated and that the polymerization is highly controllable, controlled radical polymerization allows precision macromolecular engineering (*33*). Given that commercial electron-rich monomers (e.g., styrene) are widely used in controlled radical polymerizations, we hypothesized that, if new TSCT polymers can be designed by growing polymer chains with varied end groups (as CT donor) from a single fluorophore (as CT acceptor), tunable multicolor emission might be achieved by precisely engineering the D-A distance and D type via controlled polymerization methods, alleviating the need for tedious organic syntheses.

**Fig. 1:**
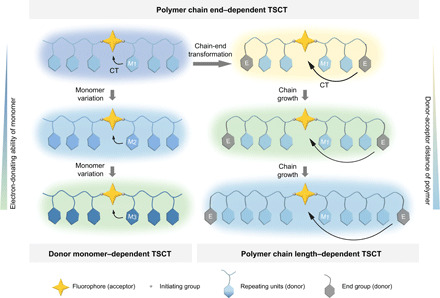
Schematic illustration of color-tunable solid-state emission from a single fluorophore by polymerization-mediated TSCT. Top row: Making chain end groups CT donors through a chain-end transformation shifts emission color from blue to yellow. Left column: Multicolor emission (blue to green) due to donor monomer–dependent TSCT. Right column: Multicolor emission (yellow to blue) due to D-A distance–dependent TSCT. M1, M2, and M3 represent the styrenic monomers with increasing electron-donating ability, and E represents transformed chain end group. The background colors represent tunable fluorescence emission colors from the polymers in aggregate/solid state. The black arrows represent both intrachain and interchain TSCT.

Here, we report a general strategy for color-tuning the emission spectrum of a single fluorophore in solid state using controlled radical polymerization–mediated TSCT. As illustrated in Fig. 1, using a library of well-defined styrenic donor (D) polymers grown from an acceptor (A) fluorophore by ATRP, we reveal that the solid-state emission color can be tuned in three different ways: (i) varying the monomer substituent, (ii) transforming the polymer end groups, and (iii) changing the polymer chain length. First, as shown in the left side of Fig. 1, the emission color can change from blue to green by increasing the electron-donating ability of the monomers, due to the TSCT from the repeating units (CT donor) to the fluorophore (CT acceptor). Second, taking advantage of an efficient end-group transformation, the polymer end groups become the CT donors, inducing a fluctuating TSCT process that results in a blue-to-yellow emission color shift. Last, as shown on the right side of Fig. 1, this TSCT process can be further manipulated by precisely tailoring the distance between the donor and acceptor, leading to tunable yellow-to-blue multicolor emission in solid state. In addition, all of the TSCT polymers are aggregation-induced emission (AIE) active (*34*–*36*). Experimental and theoretical investigations were performed to support the proposed mechanisms. We further demonstrate that our polymers, because of their simple chemical composition, can be readily processed into thin films allowing versatile photolithography.

## RESULTS

### Donor monomer–dependent emission color

**Fig. 2:**
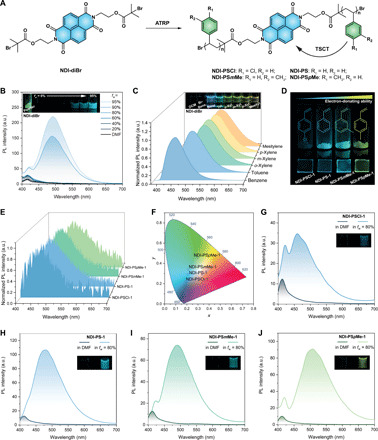
Chemical structures and photophysical properties of functionalized NDI fluorophore and NDI polymers. (**A**) Chemical structures of **NDI-diBr** and NDI polymers and representation of ATRP-mediated TSCT. The curved arrow represents both intrachain and interchain TSCT. (**B**) PL spectra of **NDI-diBr** in DMF/H_2_O mixtures. Inset: Photographs under UV lamp (365 nm) of **NDI-diBr** in powder form, as well as dissolved to a concentration of 0.1 mM in DMF/H_2_O mixtures with increasing water fraction [*f*_w_ = 0 to 95% (v/v)]. a.u., arbitrary units. (**C**) PL spectra of **NDI-diBr** in various aromatic solvents. Inset: Photographs under UV lamp (365 nm) of **NDI-diBr** in various solvents with 1.5 mM. (**D**) Photographs of powders and thin films of polymers made from different monomers. (**E**) PL spectra and (**F**) CIE 1931 chromaticity diagram of **NDI-PSCl-1**, **NDI-PS-1**, **NDI-PS*m*Me-1**, and **NDI-PS*p*Me-1** thin films. (**G** to **J**) PL spectra of the four polymers in DMF and DMF/H_2_O (20/80, v/v). Inset: Photographs of polymers in DMF or DMF/H_2_O under UV lamp (365 nm). The measuring concentration of **NDI-diBr** or NDI polymers was 5.0 μM in (B) and (G) to (J) and 1.5 mM for (C). The excitation wavelength was 370 nm. Note that curves of **NDI-PSCl-1** and **NDI-PS*p*Me-1** in (E) were smoothed by the Savitzky-Golay method. Photo credit: Suiying Ye, ETH Zurich.

Naphthalene diimide (NDI) was selected as the fluorophore for our single-fluorophore–conjugated polymers because it has a highly efficient CT-induced emission (e.g., J-aggregate and H-aggregate) and is easily derivatized (*37*). As a functional ATRP initiator with bidirectional initiating groups, **NDI-diBr** was synthesized under mild conditions in high yield (>75% yield over two steps), enabling controlled growth of polymer chains from a single NDI molecule in the center (Fig. 2A and fig. S1).

**NDI-diBr** exhibits strong yellow photoluminescence (PL) in solid state but very weak blue emission when dissolved in common organic solvents like dichloromethane (DCM) and *N*,*N*-dimethylformamide (DMF) (Fig. 2, B and C). In an aggregated state, for example, when suspended in DMF/H_2_O (20/80, v/v), **NDI-diBr** demonstrates a bright blue-green emission (λ_max_ ~ 492 nm; Fig. 2B), which indicates that **NDI-diBr** has notable AIE activity. The AIE of NDI results from the restriction of intramolecular motion that has been suggested as the working mechanism for many systems (*34*, *35*), which was also supported by the marked increase of fluorescence intensity by four orders of magnitude at low temperature (77 K; fig. S2A). Notably, the solid powders and aggregates in DMF/H_2_O differ in color, which is likely due to the different packing modes of the NDI scaffold (*37*–*39*). The J-type π-π stacking in powders, which was confirmed by single-crystal x-ray diffraction and spectrophotometry (fig. S3), is commonly reported in NDI systems (*38*). As seen in Fig. 2C, dissolved **NDI-diBr** shows enhanced emission with a shift to red in electron-rich aromatic solvents. The degree of the red shift correlates positively with the electron-donating ability of the solvent molecules (benzene < toluene < xylene < mesitylene, as indicated in table S1). This is due to the formation of CT complexes or exciplex (*37*–*39*).

We hypothesized that if a polymer chain with similar donor groups to these aromatic solvent molecules can be grown from an NDI fluorophore acceptor, a TSCT process may occur between the donor and acceptor groups in the aggregated state where they can be spatially proximal to each other. Subsequently, the emission color of the polymer may be tuned by varying the electron-donating ability of the donor group (the monomer used for the polymerization).

To test this hypothesis, using **NDI-diBr** as the initiator, a library of well-defined single-fluorophore polymer conjugates was synthesized by ATRP based on styrenic monomers with varying electron properties. The latter included *para*-chlorostyrene (SCl), styrene (S), *meta*-methylstyrene (S*m*Me), and *para*-methylstyrene (S*p*Me) (Fig. 2, A and D, table S2). As shown in table S3, four groups of NDI polymers were prepared from these monomers with varied degrees of polymerization (DPs) (mostly DP = ~20 to 40) and low polydispersity indices (Đ = ~ 1.1). All of them—NDI-poly(chlorostyrene) (**NDI-PSCl**), NDI-poly(styrene) (**NDI-PS**), NDI-poly(*meta*-methylstyrene) (**NDI-PS*m*Me**), and NDI-poly(*para*-methylstyrene) (**NDI-PS*p*Me**)—showed distinct blue-to-green emission in solid state, with maximum wavelengths ranging from ~460 to ~510 nm, independent of their molar mass (Fig. 2, D to F, and table S3). This is consistent with the fluorescence emission of **NDI-diBr** in aromatic solvents (Fig. 2C). The polymer’s fluorescence emission in solid state is the result of a TSCT process between the polymeric phenyl groups (donor) and NDI fluorophore (acceptor), as illustrated in Fig. 2A. Depending on the electron-donating abilities of the monomers (donor) used (*40*–*42*), the corresponding polymers have different TSCT states. Through-space conjugation has been used to design highly emissive molecular materials with intramolecular CT (*43*, *44*). On the other hand, TSCT can effectively be introduced to polymeric systems to generate multicolored emissions, usually based on two types of fluorophores incorporated to synthetic monomers as donor and acceptor, respectively (*12*–*14*). Distinct from previous TSCT polymers, in our system, the conventional monomers (repeating units) can directly serve as donor groups, which allows tunable solid-state emission color from an acceptor-type fluorophore.

Furthermore, all our polymers exhibited notable AIE activity. As shown in Fig. 2 (G to J), their fluorescence was much stronger in DMF/H_2_O (20/80, v/v) than in DMF. In addition, the quantum yield of the polymers in solid state increased with molar mass (table S3), which is likely caused by the stronger restriction of molecular motion of the fluorophore in longer polymer chains (*45*, *46*). The polymers that aggregated in DMF/H_2_O (20/80, v/v) maintained the same emission wavelength in solid state (e.g., thin films). We also obtained the fluorescence spectrum of **NDI-PS-1** in 2-methyltetrahydrofuran at 77 K (fig. S2B). A dramatic intensity increment was achieved at low temperature due to the AIE activity, but no emission wavelength shift was observed. While the intramolecular motion is restricted at low temperature, the polymers are still in a dilute state, and hence, the TSCT cannot take place at this D-A distance.

### Donor shifting–induced multicolor emission

**Fig. 3:**
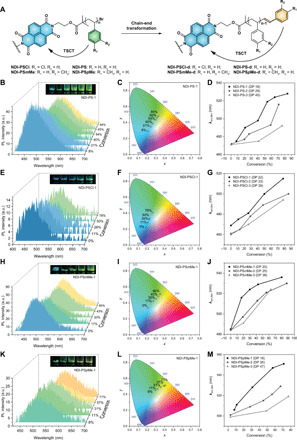
NDI polymers with high end-group fidelity (>90%) before and after debromination and their photophysical properties. (**A**) Structural illustration of end-group transformation–induced TSCT of the debrominated polymers (**NDI-PS-d**, **NDI-PSCl-d**, **NDI-PS*m*Me-d**, and **NDI-PS*p*Me-d**). The curved arrows represent both intrachain and interchain TSCT. (**B**, **E**, **H**, and **K**) PL spectra of thin films produced with (B) **NDI-PS-1**, (E) **NDI-PSCl-1**, (H) **NDI-PS*m*Me-1**, and (K) **NDI-PS*p*Me-1** (DP 22, 18, 20, and 19, respectively) before and after debromination at various conversions. Inset: Photographs under UV lamp (365 nm) of polymers in powder form with increasing debromination levels from left to right. The excitation wavelength was 370 nm. (**C**, **F**, **I**, and **L**) CIE 1931 chromaticity diagram of thin films from (C) **NDI-PS-1**, (F) **NDI-PSCl-1**, (I) **NDI-PS*m*Me-1**, and (L) **NDI-PS*p*Me-1** before and after debromination at various conversions. (**D**, **G**, **J**, and **M**) Maximum emission wavelength (λ_em_) of thin films made of NDI polymers with increasing molar masses, before and after debromination at various conversions. Note that curves of (E) and (K) were smoothed by the Savitzky-Golay method. Photo credit: Suiying Ye, ETH Zurich.

A green-yellow emission was unexpectedly observed in the context of long polymerization times (>16 hours) during the synthesis of **NDI-PS-1** (DP = 18), instead of the expected color, blue. This was not observed for polymers with higher molar mass (e.g., DP = 40). Careful revisitation of the polymer characterization data revealed the occurrence of a strong debromination side reaction when polymerization was prolonged, which was also reported in a previous ATRP study (*47*). Our data demonstrate that the original end group 1-bromoethyl benzene is transformed into vinyl benzene for some polymer chains (Fig. 3A). This transformation could have a substantial impact on the CT and subsequently the solid-state emission color of the polymers.

To elucidate this end-group effect, we performed a debromination reaction (end-group conversion) on **NDI-PS-1** with high end-group fidelity (>90%). The polymer was dissolved in DMF and heated at 100°C for varying time intervals to obtain different conversions of debromination, while the molar mass of the polymer was not affected by the reaction. This was confirmed by proton nuclear magnetic resonance (^1^H NMR) spectroscopy, gel permeation chromatography (GPC), and matrix-assisted laser desorption/ionization–time-of-flight mass spectrometry (fig. S4, A to C) (*47*). Upon increasing the amount of vinyl benzene end groups in **NDI-PS-1** (DP = 18), the bright blue emission in solid state significantly changed to green-yellow as shown in Fig. 3 (B and C). Solid-state PL spectra of thin films produced with these polymers showed that the maximum emission wavelength shifted from 475 to 528 nm (Fig. 3D). This suggests that a new TSCT state was generated because the newly formed vinyl benzene end group is a more efficient donor, which promotes CT to the NDI fluorophore. As a result, the original TSCT state was replaced in the debrominated polymers. Furthermore, the change in emission wavelength was probably not caused by variations in molecular packing or formation of NDI J- or H-aggregates because the polymers are completely amorphous, as indicated by a powder x-ray diffraction analysis (fig. S4, D and E). This was also confirmed by ultraviolet-visible (UV-Vis) absorbance measurements of the thin films, which showed similar signals in the characteristic NDI region (~360 and ~380 nm) both before and after debromination. In contrast, red shifts at the tail end of the polymers’ absorption spectra were observed after debromination (fig. S5, A to D). In addition, we did not observe the TSCT emission of NDI polymers in nonaromatic organic solvents even with concentration as high as 0.5 mM, but only a weak emission peak appeared due to the π-π stacking of the NDI core as the polymer chains are flexible enough in these solvents (fig. S6), which excluded the solvatochromism effect. These results suggest that the TSCT can only occur in aggregate/solid state in the presence of a specific CT donor.

In general, to enable an efficient TSCT process, donor and acceptor should be in close proximity (*12*–*14*, *48*, *49*). However, the donor and acceptor in our polymers (i.e., debrominated polymers) are not structurally adjacent to each other as in other reported TSCT polymer systems (*12*–*14*). This should be due to that the solid or aggregated state may allow for spatial proximity even when the donor and acceptor are far from each other along the polymer backbone. To our knowledge, there are no other such multicolor emissive systems reported to date that enables TSCT with a structurally remote D-A structure. We hypothesized that the degree of the TSCT process can be manipulated by precisely engineering the molecular D-A distance, i.e., the polymer chain length, thereby achieving tunable multicolor emissions.

To validate our second hypothesis, we studied the emission behavior before and after debromination of three different NDI-PS polymers with increasing molar masses [**NDI-PS-1** (DP = 18), **NDI-PS-2** (DP = 29), and **NDI-PS-3** (DP = 45)]. The red shift of the solid-state emission after debromination became less pronounced with increasing polymer chain length that further expands the D-A distance. The maximum emission wavelengths are 528 nm (**NDI-PS-1d**), 516 nm (**NDI-PS-2d**), and 492 nm (**NDI-PS-3d**) at the highest end-group conversion (~70%) (Fig. 3D). The distinct emission color from the three debrominated NDI-PS polymers can be seen by the naked eye (Fig. 3 and fig. S5E). This demonstrates the polymer chain length, in correlation to D-A distance, to be a key controlling factor of polymer emission color. This further suggests that TSCT degree is strongly dependent on D-A distance.

We also compared the emission properties of **NDI-PSCl**, **NDI-PS*m*Me**, and **NDI-PS*p*Me** in solid state before and after debromination by the same methods (as above). Although all polymers displayed multicolor emission after debromination, the degree of the red shift depended on the monomer substituent. As shown in Fig. 3 (E to M), debrominated **NDI-PS*p*Me-1** demonstrated much deeper yellow emissions (551 nm) compared to debrominated **NDI-PS*m*Me-1** (536 nm), while debrominated **NDI-PSCl-1** only emitted green light (515 nm), even at the highest possible debromination level. This is likely due to the different CT states caused by vinyl aromatic end groups having varying electron-donating properties. Similarly, when the different types of higher–molar mass polymers were debrominated, the significance of emission red shift decreased with the increment of polymer chain length. This is in line with the results obtained for NDI-PS (Fig. 3, D, G, J, and M; figs. S7 and S8; and table S3). In addition, a longer fluorescence lifetime was observed for the debrominated polymers relative to that of their parent polymers. This was likely due to variation in the CT process during the end-group transformation (fig. S9 and table S4). All debrominated polymers also demonstrated typical AIE behavior (figs. S10 to S12). We also obtained the fluorescence spectrum of **NDI-PS-1d** in 2-methyltetrahydrofuran at 77 K, which showed similar result to **NDI-PS-1** under the same conditions (fig. S2C). Furthermore, we reduced **NDI-PS-1** with tributyltin hydride and obtained the hydrogenated polymer **NDI-PS-1h**, which showed nearly identical emission to the former, suggesting that the Br atom at the polymer chain end has negligible impact on the fluorescence properties (fig. S13).

### Multiscale understanding from quantum chemical calculations

To elucidate the mechanism responsible for the observed multicolor emissions, we explored the electronic structures and transitions of isolated molecules and their aggregates by performing a multiscale molecular modeling study. First, we performed density functional theory (DFT) calculations to examine the effect of changes to structure on the molecular orbitals (MOs) that are potentially involved in the transitions. A number of original and fully debrominated NDI oligomers were considered using the general formulae, **NDI-PM**_**n**_ and **NDI-PM**_**n**_**-d**, respectively, where M is the monomer (SCl, S, S*m*Me, or S*p*Me), and *n* is the number of repeating units ranging from 2 to 8.

**Fig. 4:**
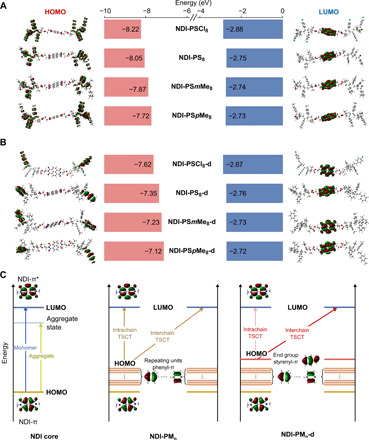
Atomistic understanding of the TSCT mechanism in the debrominated oligomers (NDI-PM_n_-d). (**A**) Comparison of spatial distribution and band diagrams of ground-state HOMO/LUMO levels for the original **NDI-PM**_**n**_ oligomers with *n* = 8—including **NDI-PSCl**_**8**_, **NDI-PS**_**8**_, **NDI-PS*m*Me**_**8**_, and **NDI-PS*p*Me**_**8**_—and (**B**) their debrominated counterparts **NDI-PM**_**n**_**-d** calculated using the CAM-B3LYP hybrid functional (*50*). By varying the monomer substituents, the HOMO-LUMO energy gap ranks by PSCl > PS > PS*m*Me > PS*p*Me, agreeing with the experimental trend. Moreover, after debromination, the HOMO levels change from backbone phenyl groups to the vinyl benzene end groups, resulting in a chain length–dependent emission red shift. (**C**) Schematic energy diagrams for the neat NDI core (left), the original **NDI-PM**_**n**_ (middle), and the debrominated **NDI-PM**_**n**_**-d** oligomers (right), respectively.

Figure 4 and figs. S14 to S18 display the calculated spatial distribution and band diagrams of ground-state MOs in the NDI oligomers considered using the CAM-B3LYP functional (*50*). We found that the lowest unoccupied MO (LUMO) always corresponds to the π*-orbital of the NDI core, while the highest occupied MOs (HOMOs) exhibit a major difference between **NDI-PM**_**n**_ and **NDI-PM**_**n**_**-d** oligomers. In **NDI-PM**_**n**_, the HOMO level is uniformly distributed on the π-orbitals of aromatic rings at the oligomer backbone. For **NDI-PM**_**n**_**-d**, the HOMO level moves instead to the π-orbital of the vinyl benzene end groups. While individual monomer moieties on the polymer backbone tend to share a similar energy level, the presence of conjugated aromatic derivatives raises the HOMO level as compared to the aliphatic counterparts (*51*). Detailed assignment of ground-state orbital energy levels for **NDI-PM**_**n**_ and **NDI-PM**_**n**_**-d** oligomers can be found in fig. S18A.

On top of spatial separation, the HOMO levels can also be fine-tuned by varying the monomer composition. The computed band diagrams in Fig. 4 (A and B) reveal that, while the LUMO levels in **NDI-PM**_**n**_ and **NDI-PM**_**n**_**-d** oligomers are almost constant, the HOMO levels are influenced by the electron-donating/-withdrawing characteristics of the monomer substituents, which rank as **PSCl** < **PS** < **PS*m*Me** < **PS*p*Me**. In other words, the HOMO-LUMO gap follows the trend of **PSCl** > **PS** > **PS*m*Me** > **PS*p*Me**, which is in line with the experimental observations discussed earlier (Fig. 3).

While the above analysis based on orbital energy levels quantitatively agree with the experimental observations, we note that the probability of electronic transition between the donor groups and the NDI core is very low due to virtually no spatial orbital overlap within isolated molecules (fig. S18B). Such effect explains the absence of CT emission in the experimental PL spectra of diluted polymer solutions (Fig. 2, G to J), where electronic transition is dominated by NDI core orbitals. We speculate that the electronic transition can occur in aggregate state due to the spatial overlap of the donor and acceptor groups in polymer coiled structures. Figure 4C presents our hypothetical mechanism for TSCT in the NDI oligomers. The energy levels of the neat NDI core (e.g., **NDI-diBr**), the **NDI-PM**_**n**_, (original), and the **NDI-PM**_**n**_**-d** (debrominated) oligomers are schematically shown, respectively. The characteristic excitation of the neat NDI core (Fig. 4C, left) corresponds to the π ➔ π* transition. Upon molecular packing, the emergence of aggregate states below the LUMO level results in an emission red shift (*38*) compared with its monomer form (Fig. 4C, left). Similarly, the optical properties of **NDI-PM**_**n**_ and **NDI-PM**_**n**_**-d** polymers are the combined effects of intra- and interchain TSCT processed. For the original **NDI-PM**_**n**_ (Fig. 4C, middle), the HOMO level is distributed on the oligomer backbone with a wide dispersity. In the molecular aggregate of **NDI-PM**_**n**_, we expect that the HOMO-LUMO transition can occur between any phenyl group surrounding the NDI core, with similar probabilities for intra- and interchain TSCT processes. On the other hand, for debrominated **NDI-PM**_**n**_**-d** polymers (Fig. 4C, right), due to the random coil structure of the polymer chains in the aggregated state and π-π stacking, a short interchain D-A distance can be more effectively achieved (fig. S19). Therefore, we infer that the interchain TSCT process is more favored for **NDI-PM**_**n**_**-d** polymers due to the higher possibility of spatial proximity of donor and acceptor groups, which explains why the red shift can still be observed even with more than 40 repeating units. With regard to long-range CT that has been observed in other systems such as double-stranded DNA (*52*), there is a lack of evidence that a similar process occurs in the polymers described here.

**Fig. 5:**
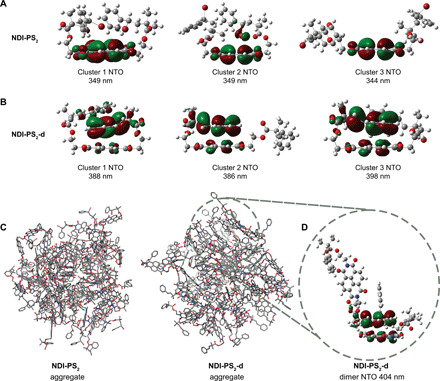
Mechanistic investigations using theoretical calculations. (**A** and **B**) **NDI-PS**_**2**_ and **NDI-PS**_**2**_**-d** originating NTOs and corresponding wavelengths for the first excited state of molecular conformations from the top three populated clusters in DMF/H_2_O (20/80, v/v) from MD simulations. (**C**) Snapshots from MD simulations of **NDI-PS**_**2**_ and **NDI-PS**_**2**_**-d** aggregates. (**D**) Dimer configuration, orbital, and corresponding wavelength of **NDI-PS**_**2**_**-d** aggregate. In all cases, transitions were to the LUMO, which was identical in spatial distribution to the LUMO of the NDI core.

To verify our assumption of aggregation-induced TSCT process in **NDI-PM**_**n**_**-d** systems, we conducted a preliminary multiscale modeling study involving molecular dynamics (MD) simulations followed by time-dependent DFT (TD-DFT) calculations to elucidate the effect of debromination on the molecular conformation (see Methods). We investigated two oligomers, **NDI-PS**_**2**_ and **NDI-PS**_**2**_**-d**, in their energy-minimized morphologies in DMF-H_2_O (20/80, v/v) and as amorphous aggregates. In both the medium (Fig. 5, A and B) and aggregate (Fig. 5C) systems, we found that the **NDI-PS**_**2**_**-d** molecules predictably stacked with their vinyl benzene rings lying above the NDI cores. TD-DFT calculations were used to further examine the MOs and optical properties of these systems based on representative conformations from the MD simulations. For isolated molecules, the excitation with the greatest oscillator strength was primarily caused by π ➔ π* transitions on the NDI core for both **NDI-PS**_**2**_ and **NDI-PS**_**2**_**-d**, with wavelengths of 344 to 354 nm (fig. S20). For **NDI-PS**_**2**_**-d**, there was an additional red-shifted transition not observed with **NDI-PS**_**2**_. This was characterized by CT from the vinyl benzene to the NDI core. A similar intermolecular configuration was observed for adjacent molecules in the **NDI-PS**_**2**_**-d** aggregate (Fig. 5D) but not in the **NDI-PS**_**2**_ aggregate.

While our calculations cannot account for all features in the experimental spectra because of the lack of explicit treatment of proximal molecules or polymer chain length, together, these results support the hypothesis that the observed red shift in emission is due to TSCT from the terminal vinyl benzene to the NDI core. It stands to reason that the CT has reduced efficiency for polymers with longer chain length due to the lower probability of the vinyl benzene groups approaching the NDI core. While the multiscale theoretical framework can be readily scaled up to simulate for even longer NDI oligomer systems, such calculations are more computationally expensive and require further force field development and validation and thus are not covered within the scope of this article.

### One-step polymerization-mediated multicolor emission

**Fig. 6:**
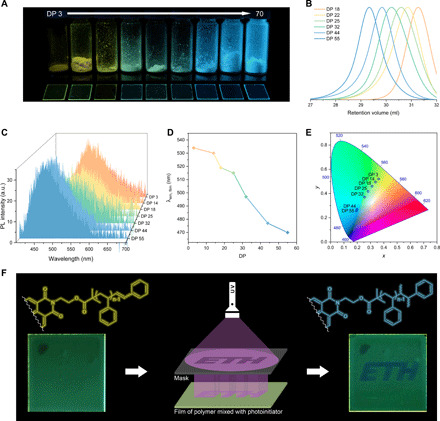
Photophysical properties of NDI-PS polymers with different DP values and low end-group fidelity (<60%). (**A**) Photographs under UV lamp (365 nm) of powders and thin films produced with polymers synthesized by ATRP with polymerization times >16 hours and DP values ranging from 3 to 70. (**B**) GPC spectra of polymers with DP 18 to 55 (polymers with DP 3 and 14 are not shown here because of the short retention time on GPC). (**C**) PL spectra and (**E**) CIE 1931 chromaticity diagram of thin films produced from **NDI-PS** with different molar masses (the polymer with DP 22 is not shown because of debromination conversion lower than 30%). (**D**) Relationship between film emission wavelength and DP of the polymers. The excitation wavelength was 370 nm. (**F**) Schematic illustration of the photolithography and photographs before and after UV irradiation of polymer thin films that contain BAPO as the photoinitiator and were produced with **NDI-PS-1d** polymers (debrominated **NDI-PS-1** at conversion 84%). Glass substrate: 24 mm by 24 mm. Photo credit: Suiying Ye, ETH Zurich.

We found that the emission wavelength of the polymers can already reach notable red shift with only 40 to 50% debromination conversion. This might be due to the presence of two end groups in each polymer chain. Therefore, multicolor emission via D-A distance–dependent TSCT might simply be achieved by synthesizing polymers with a high amount of vinyl benzene end groups in one step. To further investigate the impact of chain length on the emission color of our polymers, a series of **NDI-PS** with molar mass ranging from 1.0 to 8.0 kg mol^−1^ (DP = 3 to 70) was synthesized by ATRP with polymerization time exceeding 16 hours (versus previous condition of <3 hours, see NMR in fig. S21A). Polymers displaying multicolor emissions were obtained by varying the monomer/initiator feeding ratio, as illustrated in Fig. 6 (A to E), fig. S21, and table S5. The debromination side reaction, considered as unfavorable in conventional ATRP syntheses (*47*), facilitates a single-step provision of multicolor emission properties in our systems. Notably, the tunable emission color originates from continuous wavelength red shift, rather than the combination of multiple emission peaks. To the best of our knowledge, this has never previously been observed in simple polymer systems.

In solid or aggregated state, the maximum emission wavelength of one-step–synthesized polymers relates near linearly to polymer chain length in the range of DP 14 to 44 (Fig. 6D and fig. S21, C and F). This indicates that emission color can be precisely manipulated by controlling polymer molar mass. Note that the need for polymer chains to be narrowly dispersed may be crucial to achieve controllable fluorescence properties. This has been reported in conjugated polymer systems, which rely on more sophisticated syntheses (*53*, *54*). From DP 44 upward, the polymer emission color became insensitive to chain length variation due to the large D-A distance. This is a simple means to achieve multicolor emission from a single solid-state fluorophore by tuning polymer chain length. In addition, the polymer growth also enhanced the emission intensity, as confirmed by the absolute PL quantum yield results (table S5).

### End-group manipulation

To further exploit the end-group effect of our system and to evaluate the fluorescence variation of debrominated NDI polymers when the vinyl groups are converted into saturated groups, a simple photolithography experiment was performed. A thin film was prepared with a mixture of debrominated **NDI-PS-1d** and a commercial BAPO [phenylbis(2,4,6-trimethylbenzoyl) phosphine oxide] photoinitiator (Irgacure 819) by drop-casting onto a glass substrate (Fig. 6F). The resulting film fluoresced yellow under UV light (Fig. 6F, left). An opaque mask with hollow patterns was then placed above the thin film, followed by irradiation with a UV light source on top of the mask for several minutes (Fig. 6F, middle). This subjected the irradiated parts of the polymer thin film to a photocrosslinking reaction, resulting in conversion of the vinyl groups into saturated bonds. Due to the transformation, the UV-irradiated parts devoid of vinyl groups fluoresced blue under UV, which is consistent with the emission color of **NDI-PS-1** (original polymer, before debromination). The parts protected from irradiation by the mask remained yellow (Fig. 6F, right). PL spectra were recorded on the thin films produced with **NDI-PS-1d** and 5% BAPO mixture before and after UV irradiation (fig. S22), wherein the latter shows a considerable blue shift (λ_max_ from ~530 to ~480 nm). This confirms the effect of polymer end groups on emission color in single-fluorophore–based TSCT polymer systems and provides a new and versatile photolithography strategy. Moreover, adapted from a reported method (*55*), we attached cinnamyl and naphthalene group to the chain end of **NDI-PS-1** by postfunctionalization with cinnamyl alcohol and 1-naphthaleneethanol in the presence of AgClO_4_, respectively. As expected, the resulted polymers with new electron-donating end groups exhibited new emission peaks with red shift, which further confirmed the end-group engineering versatility of this polymer system (fig. S23).

## DISCUSSION

In this work, we synthesized a library of conventional and substituted polystyrenes (as CT donor) with varied molar masses using functionalized NDI (as CT acceptor)–initiated ATRP. The obtained polymers exhibited typical TSCT properties. Under specific reaction conditions or through a direct polymerization process, the chain end groups of the polymers can become the CT donor, inducing an unexpected fluctuation of the TSCT process that results in a continuous emission color change. Taking advantage of the controlled radical polymerization, we demonstrated that the solid-state emission color of this acceptor-type single fluorophore can be readily tailored by precision macromolecular engineering of the polymer as follows:

1) Monomer substituent variations: TSCT from the repeating units to the fluorophore can be modulated by varying the electron-donating ability of the donor. This can simply be achieved by selecting commercially available monomers with different substituents. This kind of engineering enabled emission colors from blue to green (λ_max_ from ~460 to ~510 nm) for NDI polymers upon increasing the electron-donating ability of the monomer. More electron-donating monomers with different structures may be used to further modulate the emission color.

2) End-group transformation: TSCT can occur between the newly generated polymer chain end group and the fluorophore, even when they are not adjacent in molecular structure. This enabled an emission color shift from blue to yellow (e.g., λ_max_ of **NDI-PS-1** ranges from ~475 to ~530 nm) for the NDI polymers. Notably, new-type end groups may be introduced by specific reactions to further manipulate the emission color, thanks to the efficient end-group transformation enabled by controlled radical polymerization.

3) Chain length changes: Benefiting from the controllability of ATRP, end-to-core TSCT can be obtained by precisely tailoring polymer chain length and with it the distance between the polymer chain end-group CT donor and the fluorophore CT acceptor. Yellow-to-blue emission colors (λ_max_ from ~540 to ~470 nm) were obtained for the NDI polymers. Because of the distinct structure of different polymer systems, other polymers can also be designed to further explore the chain length–dependent emission color (e.g., aromatic methacrylates).

Theoretical calculations presented in this study support the proposed color-tuning mechanisms. In addition, a simple photolithography experiment was performed, which confirmed that the polymer end group plays a crucial role in tuning the emission color of single-fluorophore polymer conjugates. All monomers used in this study are commercially available, which further highlights the simplicity of our developed system since there is no need to prepare functionalized monomers via multistep syntheses to obtain tunable emission. The D-A distance can be tuned simply by changing polymer chain length, avoiding complex molecular designs. Because of the versatility of our system, the usage of other fluorophores, monomers, and chain end groups can be exploited to develop polymers with emission ranges all over the color spectrum. Our methodology can also be used with other controlled polymerization techniques, such as nitroxide-mediated polymerization, reversible addition-fragmentation chain transfer polymerization, and ring-opening polymerization, to design color-tunable emissive polymers. Overall, this work proposes an unprecedented yet simple strategy to develop single-fluorophore–based multicolor emissive solid materials for various application areas.

## METHODS

### Synthesis of NDI-diBr

The synthesis, adapted from a previously described procedure (*56*), was conducted in two steps. First, 1,4,5,8-naphthalenetetracarboxylic dianhydride (2.68 g, 10.0 mmol) and ethanolamine (3.06 g, 50.0 mmol) were dissolved in 150 ml of ethanol. After stirring at 90°C overnight, the mixture was poured into deionized water (200 ml), and the precipitate was washed with ethanol (100 ml). The NDI with dihydroxyl groups (**NDI-diOH**) was obtained as a light yellow solid. Subsequently, **NDI-diOH** (2.80 g, 8.0 mmol) was dispersed in 65 ml of pyridine and 10 ml of tetrahydrofuran (THF) under argon. A solution of α-bromoisobutyryl bromide (8.92 g, 40.0 mmol) in 15 ml of THF was slowly added to the mixture. After stirring at room temperature overnight, the solution was poured into deionized water (200 ml), and the precipitate was filtered and washed with ethanol (100 ml). The light brown solid obtained was further purified by column chromatography on silica gel with DCM as the eluent. The NDI with dibromoisobutyryl groups (**NDI-diBr**) was obtained as a pink solid (3.97 g, 76% over two steps). ^1^H NMR [400 MHz, chloroform-d (CDCl_3_)]: parts per million = 1.86 (s, 12H, -C*H*_3_-), 4.58 (m, 8H, -C*H*_2_-), and 8.77 (s, 4H, aromatic ring). ^13^C NMR (75 MHz, CDCl_3_): δ = 171.6, 162.8, 131.1, 126.8, 126.6, 63.1, 55.7, 39.3, and 30.7. High-resolution mass spectrometry (ESI+): *m/z* (mass/charge ratio)= 652.9964 (M + H)^+^.

### Synthesis of NDI polymers

All polymer syntheses were carried out by **NDI-diBr**–initiated ATRP. Different DPs were achieved either by varying the monomer/initiator feeding ratio or by controlling the reaction time. In a typical procedure, 1.0 equivalent (eq.) of **NDI-diBr** was loaded in a dry Schlenk flask, along with copper(I) bromide (CuBr; 1.5 eq.) and 4,4′-dinoyl-2,2′-dipyridyl (1.5 eq., except **NDI-PS*p*Me-2** and **NDI-PS*p*Me-3**). The Schlenk flask was evacuated and refilled with argon through several cycles to remove oxygen. In the meantime, the monomer was deoxygenated by bubbling argon for at least 20 min. For **NDI-PS*p*Me-2** and **NDI-PS*p*Me-3**, the monomer was mixed first with 1,1,4,7,7-pentamethyldiethylenetriamine (PMDETA; 1.5 eq.) and degassed with argon. Desired amounts of the degassed monomer (or mixture with PMDETA) were added to the Schlenk flask using a syringe under argon. The Schlenk flask was then heated at 115°C (for **NDI-PS-1**) or 80°C (for others) in an oil bath. After a specific time, the Schlenk flask was taken out of the oil bath and cooled down with water to stop the reaction. Last, the brown residue was consecutively dissolved in THF, filtrated through basic alumina column, precipitated once in hexane and twice in methanol, and then dried under vacuum overnight. Final product was obtained and ready for further analyses. The synthesized polymers were characterized by ^1^H NMR spectroscopy (figs. S24 to S28) and GPC (fig. S30).

### Debromination of NDI polymers

In a typical procedure, 0.2 to 0.4 g of polymer with high end-group fidelity was loaded in a dry Schlenk flask, followed by several evacuation and argon-filling cycles to remove oxygen. A total of 2 ml of extra-dry DMF was degassed by bubbling argon and then was loaded to the Schlenk flask under argon. The Schlenk flask was heated at 100°C in an oil bath. To obtain different conversions of the end groups, about one-fifth of the reaction mixture was taken out from the Schlenk flask using a syringe at different time intervals (5, 10, 20, and 45 min for **NDI-PS*p*Me**; 0.5, 1, 2, 4, and 6 hours for others). The mixture was immediately cooled down, precipitated twice in methanol, and dried overnight under vacuum to yield the final product for further analyses. The debrominated polymers were characterized by ^1^H NMR spectroscopy (figs. S24 to S28), solid-state ^13^C NMR (for **NDI-PS-1** and **NDI-PS-1d**; fig. S29), and GPC (fig. S30).

### End-group engineering of NDI polymers

Hydrogenation of **NDI-PS-1** was adapted from the previously reported method (*57*). Specifically, **NDI-PS-1** (1.0 eq.) and CuBr (0.5 eq.) were loaded in a Schlenk flask and vacuum for 20 min, followed by a vacuum-argon cycle for three times. Next, extra-dry THF was loaded into the flask and deoxygenated in argon flow for 10 min, followed by loading of PMDETA (0.5 eq.) under argon. The mixture was stirred at room temperature for 30 min under argon. Tributyltin hydride (3.0 eq.) was then added into the reaction flask under argon. The flask was placed in an oil bath at 60°C for 4 hours with stirring. Last, the residue was consecutively dissolved in THF, filtrated through basic alumina column, precipitated twice in hexane, and then dried under vacuum overnight.

End-group functionalization with cinnamyl and naphthalene groups was performed according to the reported method (*55*) and specified as follows. AgClO_4_ (3.0 eq.) was loaded in a Schlenk flask; **NDI-PS-1** (1.0 eq.) and cinnamyl alcohol or 1-naphthalenemethanol (20 eq.) were loaded in another Schlenk flask, followed by an 1-hour evacuation and next a vacuum-argon cycle for three times. Then, extra-dry toluene was added to dissolve the polymer and alcohol under argon. The solution was then transferred into the first flask under argon. The flask was placed in an oil bath at 25°C under stirring overnight. Last, the residue was consecutively dissolved in THF, filtrated through basic alumina column, precipitated twice in methanol, and then dried under vacuum overnight. Final product was obtained and ready for further analyses.

### Drop-casting and photolithography process

An opaque mask with hollow patterns (logo of ETH) was designed and three-dimensionally (3D) printed using photopolymer resin (Formlabs, Clear FLGPCL02). Samples of **NDI-PS-1d** (debrominated **NDI-PS-1**) and BAPO (5.0 weight %) were dissolved in toluene to obtain a polymer solution (10 mg ml^−1^). A volume of 200 to 300 μl of the prepared solution was carefully dropped onto a 24 mm by 24 mm glass substrate, followed by low-vacuum evaporation to give a solid thin film. A photo of the obtained thin film was taken under UV. After the solvent was completely evaporated, the prepared mask was placed above the thin film, on top of which a UV light source was applied for 5 to 10 min. A photo of the thin film after photolithography was taken under UV.

### Theoretical investigations of isolated molecules and aggregates

#### MD simulations

To examine the behavior of **NDI-PS**_**2**_ and **NDI-PS**_**2**_**-d** monomers in solution, a molecule of each was individually solvated in a 50 Å by 50 Å by 50 Å box with 196 DMF molecules and 3360 water molecules to produce a DMF/H_2_O (20/80, v/v) solution. All MD simulations were performed using the GROMACS 2018 MD code (*58*). Force field parameters for DMF, **NDI-PS**_**2**_, and **NDI-PS**_**2**_**-d** were obtained from the CGenFF version 2.2.0 program (*59*) and CGenFF force field version 4.1 (*60*). Pairwise electrostatic interactions were cut off at 1.1 Å with a particle mesh Ewald treatment of long-range interactions. A switching function was used to reduce the van der Waals interactions to zero between 9 and 10 Å. Covalent bonds involving hydrogen were constrained using the LINCS algorithm. Simulations were run in the isothermal-isobaric (NPT) ensemble, with the Nosé-Hoover thermostat and Parrinello-Rahman barostat used to maintain a constant temperature of 300 K and pressure at 1.0 × 10^5^ Pa, respectively. A time step of 2 fs was used, with simulation snapshots saved every 10 ps. Following energy minimization, each system was equilibrated by linearly heating from 10 to 300 K over 1 ns. Initial MD simulations were run for 100 ns, and root mean square deviation (RMSD) clustering analysis was performed using VMD 1.9.3 (*61*) on the final 50 ns with a 50-ps time interval, resulting in 1000 snapshots for each system. The RMSD cutoff of 3 Å populated the top three clusters with at least 50% of the simulation snapshots. Representative snapshots from the top three clusters in a 2:2:1 ratio were resolvated in DMF/H_2_O (20/80, v/v) and run for three independent 100-ns runs to produce an ensemble of five-molecule aggregates. Ten separate aggregates were packed into a 70 Å by 70 Å by 70 Å box and simulated for 100 ns to produce aggregate structures comprising 50 individual molecules.

#### DFT calculations

DFT calculations were performed to investigate the excitation energies of various NDI derivatives under vacuum or implicit solvent model. All DFT calculations are carried out by quantum chemistry package Gaussian 16, Revision C.01 (*62*). For DFT calculations in vacuum, saturated NDI oligomers (**NDI-PM**_**n**_) and their debrominated counterparts (**NDI-PM**_**n**_**-d**) where M = S, SCl, S*m*Me, and S*p*Me and *n* = 2, 4, 6, and 8 were considered. The input structures of NDI oligomers were inferred from the **NDI-diBr** crystal structure and further optimized using CAM-B3LYP functional14 and 6-31+G(d,p) basis set. TD-DFT calculations were performed to extract the transition energies and orbital population using the same functional and basis set as in geometric optimization. Solvent-inclusion DFT calculations were performed on representative snapshots from MD simulations, as well as on the **NDI-diBr** x-ray crystal structure, using the 6-31G(d,p) basis set and M06-2X DFT functional for geometry optimization, with a frequency calculation to confirm the absence of negative frequencies. We used a similar modeling protocol to explore the aggregation-induced excitonic quenching of perylene diimide chromophores in poly(methyl methacrylate) (*63*). TD-DFT calculations were performed using the 6-31+G(2d,p) basis set and CAM-B3LYP DFT functional (*53*). In all cases, the polarizable continuum model (PCM) (*64*) was used to approximate the presence of water implicitly. Natural transition orbitals (NTOs) were determined for the first two excited states for each system.

## SUPPLEMENTARY MATERIALS

Supplementary material for this article is available at http://advances.sciencemag.org/cgi/content/full/7/15/eabd1794/DC1
